# Retracted: Molecular Hydrogen Effectively Heals Alkali-Injured Cornea via Suppression of Oxidative Stress

**DOI:** 10.1155/2022/9846572

**Published:** 2022-05-09

**Authors:** Oxidative Medicine and Cellular Longevity

*Oxidative Medicine and Cellular Longevity* has retracted the article titled “Molecular Hydrogen Effectively Heals Alkali-Injured Cornea via Suppression of Oxidative Stress” [1], due to concerns with the figures. The journal was contacted by a reader who identified that Figure 5(c) in [1] is duplicated with Figure 4(j) in [2] and Figure 5(a) in [3].

The authors were asked for clarification, but did not satisfactorily address the concerns of the Editorial Board. The article is therefore being retracted due to concerns regarding the reliability of the data. Authors Dr. Jitka Cejkova and Dr. Cestmir Cejka are deceased, the remaining authors agree to the retraction.
